# Highly Selective CMOS-Compatible Mid-Infrared Thermal Emitter/Detector Slab Design Using Optical Tamm-States

**DOI:** 10.3390/ma12060929

**Published:** 2019-03-20

**Authors:** Gerald Pühringer, Bernhard Jakoby

**Affiliations:** Institute for Microelectronics and Microsensors, Johannes Kepler University Linz, 4040 Linz, Austria; bernhard.jakoby@jku.at

**Keywords:** thermal emitter, Tamm plasmons, silicon photonics, mid-infrared

## Abstract

In this work, we propose and evaluate a concept for a selective thermal emitter based on Tamm plasmons suitable for monolithic on-chip integration and fabrication by conventional complementary metal oxide semiconductor (CMOS)-compatible processes. The original design of Tamm plasmon structures features a purely one-dimensional array of layers including a Bragg mirror and a metal. The resonant field enhancement next to the metal interface corresponding to optical Tamm states leads to resonant emission at the target wavelength, which depends on the lateral dimensions of the bandgap structure. We demonstrate the application of this concept to a silicon slab structure instead of deploying extended one dimensional layers thus enabling coupling into slab waveguides. Here we focus on the mid-infrared region for absorption sensing applications, particularly on the CO_2_ absorption line at 4.26 µm as an example. The proposed genetic-algorithm optimization process utilizing the finite-element method and the transfer-matrix method reveals resonant absorption in case of incident modes guided by the slab and, by Kirchhoff’s law, corresponds to emittance up to 90% depending on different choices of the silicon slab height when the structure is used as a thermal emitter. Although we focus on the application as an emitter in the present work, the structure can also be operated as an absorber providing adjusted lateral dimensions and/or exchanged materials (e.g., a different choice for metal).

## 1. Introduction

**Figure 1:**
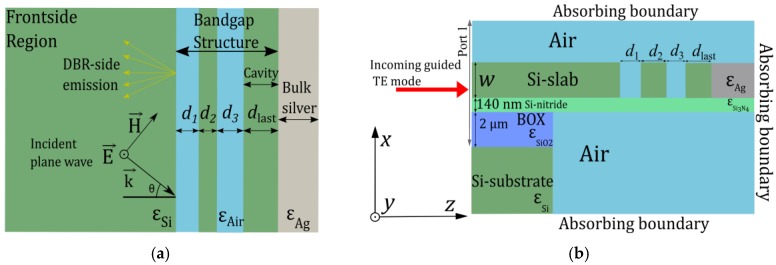
1-D Simulation domain of the conventional 1D TP structure for the transfer-matrix method (**a**) and 2-D FEM simulation domain of the STP structure (**b**). The circle with the center dot indicates the out-of-plane *y*-axis.

Selective thermal emitters gained increased attention in recent years in context with several sensing applications [1,2,3,4,5]. Particularly, so-called resonant Tamm-plasmon (TP) structures (example is illustrated Figure 1a) have been investigated extensively, as layered structures can, in principle, be fabricated using established, highly reliable deposition processes [6,7,8,9,10]. Tamm states can exist near the interface between a distributed Bragg reflector and a metal. Excitation of these states by external energy (e.g., thermal or laser pumping) leads to highly adjustable and performant emission properties. However, the integration of such layered structures to on-chip optical sensing applications is very challenging and cost-intensive, as can be seen in the implementation in [11], for example. A direct on-chip implementation can be realized by lateral patterning, which can be provided by CMOS (complementary metal oxide semiconductor) technologies. Particularly, e-beam lithography and deep reactive-ion etching together with lift-off processes facilitate the patterning of slab structures, which would enable low-cost mass fabrication of corresponding devices. This led us to the question whether a TP structure in a slab scenario can be realized and what its performance would be. Such a design promises performant infrared (IR) sources featuring monolithic on-chip integration and coupling to waveguide designs together with cheap mass-fabrication. For these reasons, slab designs are in high demand for industry. Purely one-dimensional, layered designs (1D TP structures) would require additional intricate coupling structures for in-plane guided modes, which also introduce additional coupling losses.

Although the concept of 1D TP structures have been studied extensively in the past, there are only few studies dealing with TP structures featuring one constrained spatial dimension of the multilayers perpendicular to their surface normal vector. Symonds et al. demonstrated the existence of Tamm states apart from purely 1D-layered scenarios by structuring the metal as microdiscs or as rectangles, which revealed interesting behavior of the emission properties regarding polarization dependence [12]. Also, the existence of optical Tamm states as well as surface plasmon-polaritons (SPPs) has been demonstrated in photonic crystal nanobeam structures [13].

However, to the best of our knowledge, there are no studies dealing with TP structures in a pure silicon slab scenario yet. We label the novel concept of such a resonator in the slab scenario as slab Tamm-plasmon (STP) structure. In this work, we model a STP structure (see Figure 1b) by applying concepts used for designing 1D-TP structures featuring extended layers as well as concepts for optical resonators in dielectric slabs. We focus on a design for silicon based materials, which makes the structures particularly interesting for mid-IR sensing applications where silicon is transparent. Also, expensive quantum-well materials in the dielectric region of the field enhancement (such as in [6]) are not needed in the field of thermal mid-IR emission. The near/mid IR spectral region is particularly interesting for absorption gas sensing and constitutes a huge field with great potential for novel highly-integrated devices [14,15].

The design considered in this work is based on TP emission structures featuring light emission directed along the side of the distributed-Bragg-reflector (i.e., DBR-side emission, see [7]) and bulk silver as a mirror, as can be seen in Figure 1a. In contrast, TP structures featuring emission on the metal-side (see, e.g. [8]) are not as convenient for fabrication in the slab scenario, as thin metal layers are required in this case. The optical properties of a layered 1D TP structure (Figure 1a) are determined by the optical properties of the materials (dielectrics and metals) and the stack configuration (i.e., number of layers and their thicknesses). As a first step towards a STP-structure, we design a 1D TP structure featuring unity absorptance at the target mid-IR resonance $\lambda_{0}$ wavelength using temperature models for Si and Ag.

In analogy to previous works, the emittance is enhanced to unity by variation of the individual layer thicknesses using a genetic-algorithm (GA) optimization [16,17]. Then, the structure is transformed into a slab design with finite height, which introduces light confinement by index guiding resulting in an incomplete bandgap (i.e., possibility of radiation) in the optical dispersion of the aperiodic bandgap structure. This inevitably leads to radiation losses along the surface normal of the slab, as optical resonances in slab structures always feature associated nonzero Fourier components inside the light cone [18,19]. In conclusion, the featured TP design provides vertical (in-plane) light-confinement by index guiding as well as lateral light-confinement by the optical TP state located at the DBR-metal interface, similar to defect cavities in photonic crystal slab structures [19]. The optical resonance (i.e., field enhancement near the dielectric-metal interface) features damping through lateral losses by coupling to modes in the slab waveguide and absorption in the metal as well as vertical losses by radiation. However, the degree of the light confinement inside the STP structure is limited by the properties of the corresponding Si-slab waveguide mode. That is, decreasing degrees of confinement have to be expected for decreasing slab heights (i.e., decreasing effective indices, together with increasing evanescent tails of the guided wave [20]). As a result, the lateral dimensions of the optimized STP structures for minimal radiation losses are crucially impacted by the thickness of the Si-slab. In this work, we show that high-Q resonances in STP-structures are possible for typical slab thicknesses used in mid IR Si slab-waveguides based on evolutionary optimization methods. The simulation of the complete waveguide-resonator-metal system implicates effective coupling between the guided mode in the Si-slab and the resonant TP mode, which enables monolithic on-chip integration of the obtained STP designs.

As mentioned above, we propose a two-step optimization procedure: First, the purely one-dimensional TP structure was found via GA optimization. This yielded the initial configuration for the second GA optimization in the slab scenario. Both procedures were repeated for different slab heights corresponding to different effective indices, inclination angles, etc. Four different slab heights (0.75, 1, 1.25, and 1.5 µm) and two different total number of dielectric layers (four and six) were considered for the optimizations. This set of thicknesses should be suited for being processed by semiconductor technologies. Increasing the slab thickness even further would not only exacerbate processing the slab, also an uncontrollable number of parasitic overtone resonances would emerge in the emission spectrum. The target mid-IR resonance wavelength $\lambda_{0}$ is kept constant at 4.26 µm (${\mathrm{CO}}_{2}$ absorption line), which features low absorption of silicon-based material together with high potential for sensing applications. For one-dimensional simulations, the transfer matrix method (TMM) was employed, whereas the two-dimensional structures were characterized by finite-element method simulations (both methods in frequency-domain) via the software package Comsol Multiphysics 5.3 (COMSOL Inc., Stockholm, Sweden).

The paper is structured as follows: In the following chapter, we provide some theoretical considerations necessary for the transitions from 1D TP to STP structures. Then, we set up the geometry and materials for the 1D TP structure and optimize the stack configurations with a GA algorithm. In the next chapter, we describe the application of a second GA optimization via simulations of STP structures for further optimization. We conclude with a discussion of the results and future perspectives for experimental realization. Preliminary results of our work have been published at the conference “Eurosensors 2018” [21].

## 2. Design, Methods, and Theory of STP Resonators

### 2.1. Heater Design and Material Properties

Figure 1 shows the simulation domains of the resonant structures investigated in this work, i.e., a conventional 1D TP structure in (a) and the corresponding STP structure in (b). Previous 1D TP structures mainly differed in the dielectric materials and the sequence of layers. The slab design (Figure 1b) laterally features the same structure as the 1D TP design, namely a distributed reflector (DR) as a bandgap structure including a silicon layer of width $d_{\mathrm{last}}$, which terminates at a silver interface. The silver surface acts as reflective mirror and source of radiation and absorption [22,23]. In the scenario of thermal emission, the source of thermal energy can be provided by an integrated heater next to the Ag, such as implemented in [14], or by direct Joule heating of Ag, for example. Our design includes a 140 nm thick SiN membrane, which enables straightforward integration of arbitrary, CMOS-compatible heater designs.

In analogy to the approaches in references [17,22], we employ a temperature models for the complex refractive index N=n+ik of silver and silicon in order to account for the degradation of the reflectivity of the Si-Ag interface. The temperature was set to 500 K for the silicon slab waveguide and to 800 K for the materials (Si and Ag) located on the silicon nitride membrane. This choice of the heater operation temperature ensures a high intensity of mid-IR radiation, but results in a significant modification of the optical properties of Si and Ag. The resulting values for Si at $\lambda_{0}=4.26$ µm were $N_{Si}=$ 3.46 (500 K) and $N_{Si}=$ $3.52+\;7.3\times 10^{-4}\;i$ (800 K), respectively. The corresponding refractive index for silver (800 K) was $N_{\mathrm{Ag}}=7.56+24.85i$.

### 2.2. Temporal Coupled-Mode Theory

Optically resonant slab structures can be modeled independently from their particular geometry via temporal coupled-mode theory [19,24]. This framework only considers a resonance with amplitude A, which is connected to several ports featuring individual coupling for incoming or outgoing energy. In analogy to the filter structure in a transmission scenario in [19], the evolution of A of the field resonance can be modeled via one incoming port (associated with the waveguide) and two outgoing ports (associated with radiation and metal-absorption, respectively) in a scenario of weak coupling as

(1) $$\frac{dA}{dt}=-i\omega A=-i\omega_{0}A-\frac{A}{\tau_{\mathrm{Leak}}}-\frac{A}{\tau_{\mathrm{Metal}}}-\frac{A}{\tau_{P}}+\sqrt{\frac{2}{\tau_{\mathrm{Leak}}}}a_{\mathrm{in}}\;$$

where $a_{\mathrm{in}}$ is the amplitude of the incident wave and $\tau_{i}$ are the resonance lifetimes associated with the individual damping mechanisms of the STP resonance, namely leakage into the waveguide ($\tau_{\mathrm{Leak}}$), absorption by the metal ($\tau_{\mathrm{Metal}}$) and a parasitic absorption mechanism such as intrinsic material absorption or radiation losses ($\tau_{P}$). Together with the relation between $a_{\mathrm{in}}$ and the outgoing amplitude

(2) $$a_{\mathrm{out}}=-a_{\mathrm{in}}+\sqrt{\frac{2}{\tau_{\mathrm{Leak}}}}A$$

The reflection coefficient can now be calculated with equations (1) and (2) as

(3) $$R\left(\omega \right)=\frac{{\left|a_{\mathrm{out}}\right|}^{2}}{{\left|a_{\mathrm{in}}\right|}^{2}}=\frac{{\left(\omega -\omega_{0}\right)}^{2}+{\left(\frac{1}{\tau_{\mathrm{Leak}}}-\frac{1}{\tau_{\mathrm{Metal}}}-\frac{1}{\tau_{P}}\right)}^{2}}{{\left(\omega -\omega_{0}\right)}^{2}+{\left(\frac{1}{\tau_{\mathrm{Leak}}}+\frac{1}{\tau_{\mathrm{Metal}}}+\frac{1}{\tau_{P}}\right)}^{2}}\;$$

Neglecting the effect of intrinsic absorption (i.e., $\tau_{P}\to \infty$) we obtain the regular resonance condition $\tau_{\mathrm{LM}}∶=\tau_{\mathrm{Leak}}=\tau_{\mathrm{Metal}}$ (“LM” stands for “Leak = Metal”), which results in unity absorptance for resonant 1D TP structure without parasitic damping mechanisms (i.e., all of the incident radiation is absorbed by the metal). The scenario of weak coupling now assumes an unchanged resonance condition if $\tau_{P}$ is large, but finite. Thus, it is required for $\tau_{P}$ to be significantly larger than the matched resonance lifetimes. In order to see this, we can rewrite equation (3) in terms of quality factors $Q_{i}=\frac{\tau_{i}\omega_{0}}{2}$, where *i* stands for “LM” or “P”:

(4) $$R\left(\omega_{0}\right)=\frac{{\left(-\frac{1}{2Q_{P}}\right)}^{2}}{{\left(\frac{1}{Q_{\mathrm{LM}}}+\frac{1}{2Q_{P}}\right)}^{2}}={\left(\frac{1}{Q_{P}}\right)}^{2}\cdot {\left(\frac{Q_{\mathrm{LM}}Q_{P}}{2Q_{P}+Q_{\mathrm{LM}}}\right)}^{2}={\left(\frac{Q}{Q_{P}}\right)}^{2}\cdot {\left(\frac{Q_{\mathrm{LM}}}{Q+Q_{\mathrm{LM}}}\right)}^{2}$$

In the last step, the total quality factor $Q=\frac{Q_{P}Q_{\mathrm{LM}}}{Q_{P}+Q_{\mathrm{LM}}}$ was introduced together with algebraic conversions. An analogous procedure was also applied in references [19,24] for resonant structures featuring filter transmission in nanobeams. It can be seen from this result, that the reflection is negligible if $Q_{P}\gg Q_{\mathrm{LM}}$ and $Q_{P}\gg Q$ holds. Unfortunately, this does not tell us the total amount absorbed by the metal, only that reflection is not the dominating effect for resonance damping if the above condition holds. Rather, the damping (causing deviation from unity absorptance) is dominated by the parasitic absorption mechanism such as Si-absorption or radiation losses in a slab configuration. This result suggests $Q_{\mathrm{LM}}$ should not be too high in order to suppress reflection back into the waveguide. This limits the number of dielectric layers used for the bandgap structure, as well as the reflectivity of the metal in use. On the other hand, if $Q_{\mathrm{LM}}$ is too low, the weak coupling condition does not hold and the approach of temporal coupled mode theory fails. In the STP scenario, these loss mechanisms represented by $Q_{\mathrm{LM}}$ and $Q_{P}$ have to be aligned for varying slab thicknesses *w* in order to maximize the light confinement for the STP mode for strong emission or absorption.

## 3. 1D Tamm Plasmon Structures

### 3.1. Resonance Condition for Optical Tamm States

The sequence of materials of the 1D TP structure (Figure 1a) was chosen in such a way, that the transition to a slab design is seamlessly possible. The DBR is composed of Air/Si layers starting with air, which terminate at a bulk silver layer (DBR-side emission). Similar to previous 1D TP structures previously simulated and measured, the material of the thickness-adjusted last dielectric layer ($d_{\mathrm{last}}$) is silicon [7,8]. As this structure shall be transformed into a STP configuration, the continuous region at the beginning of the domain is also composed of Si. The optimal number of layers for maximum $A_{\mathrm{STP}}\left(\lambda_{0}\right)$ can be calculated by aligning the reflectance of the DBR with the reflectance of the metal, as applied in [7,9,22], for example. Thus, the optimal number of layers depends their refractive indices and on the reflectance of the dielectric-metal interface.

As described in Section 2.1, the modification of the optical material properties of Si and Ag by the applied temperature models results in a significant modification of the reflection properties of the Si-Ag interface for elevated temperatures. This is particularly important, as this promotes leakage and metal absorption as dominating absorption mechanisms in the STP scenario ($Q_{P}\gg Q_{\mathrm{LM}}$). Together with the conventional expression for power reflectance $R={\left|r\right|}^{2}$ (r is amplitude reflection coefficient given by the Fresnel equation) the reflectance of a Si-Ag interface degrades to $R_{\mathrm{Ag}}\left(800\;K\right)∼0.85$ at perpendicular incidence. This compares to the value for room temperature $R_{\mathrm{Ag}}\left(300\;K\right)∼0.95$. This drop in reflectance has severe impact on the resonance condition for TP structures featuring DBRs, as there does not exist an integer value for a suitable number of quarter-wavelength (QW) layers *N* for satisfying the resonance condition for TP structures [22].

The condition for a undamped resonance for Tamm plasmon structures can be expressed as $r_{\mathrm{DBR}}r_{M}=1$ (see Ref. [25]), where $r_{\mathrm{DBR}}$ and $r_{M}$ denote the amplitude reflection coefficients of the metal and the DBR, respectively. A Bragg mirror composed of three dielectric QW layers with silicon as medium in the frontside (reflection) and backside (transmission) region (i.e., the configuration reads as Si-*LHL*-Si, where *L* denotes the low index QW layer and *H* the high index QW layer), features the closest approximation for resonance ($\left|r_{\mathrm{DBR}}r_{M}\right|=0.91$, $\mathrm{Arg}(r_{\mathrm{DBR}}r_{M})=25.3{}^{\circ}$) for an integer number of QW-layers. 

As this represents a distinct mismatch of the resonance condition for 1D TP structures, a mechanism to modify $r_{\mathrm{DBR}}$ is demanded for a better phase matching of the TP-eigenmode condition. Although reducing the number of layers further down to one double-layer slightly improves the phase matching ($\mathrm{Arg}(r_{\mathrm{DBR}}r_{M})\approx 18{}^{\circ}$), the high losses in reflection amplitude ($\left|r_{\mathrm{DBR}}\right|$ low) leads to a highly damped resonance not satisfying the weak coupling assumption. GA optimization allows an alignment of the multilayer stack by variation of its individual layer thicknesses in order to achieve optimal matching.

### 3.2. Simulation of 1D TP Structures

Figure 1a illustrates the simulation domain for the 1D TP structures. We simulate the situation of an incident plane wave with wave vector $\vec{k}$ at an angle θ. More details regarding the simulation process are explained in Appendix A.2. 

Reference [16] demonstrated that the *Q*-factor of TP structures can be dramatically increased by applying a GA optimization process on the individual thicknesses of the dielectric layers. Particularly, the magnitude of the improvement is determined by the number of dielectric layers *N*. However, *N* and the individual layer thicknesses $d_{i}$ (where i= 1…*N*) cannot be varied in an unlimited manner, as parasitic resonance modes appear inevitably in the spectral vicinity of $\lambda_{0}$ in an uncontrollable manner [17]. We demonstrate this concept via a GA optimization with bounds on the layer thicknesses for four and six dielectric layers in order to achieve an optimal subwavelength structure featuring an optimum matching of the resonance condition for a given $r_{M}$ resulting in higher *Q* factors and field enhancement. Unity absorptance (i.e., $A_{1D}\left(\lambda_{0}\right)=1$) together with high *Q* factors for the 1D TP structures represents the first best guess for a precondition for a justified weak coupling assumption (i.e., successful confinement of the field in slab scenario). Also, limiting the extent of the air gaps by defining appropriate constraints in optimization is also beneficial in view of the slab scenario, as a STP mode has empirically shown to be more affine to confine light in the slab if the field enhancement is located at Si sites.

### 3.3. Genetic Algorithm Optimization via 1D TP Structures in Fitness Function

**Figure 2:**
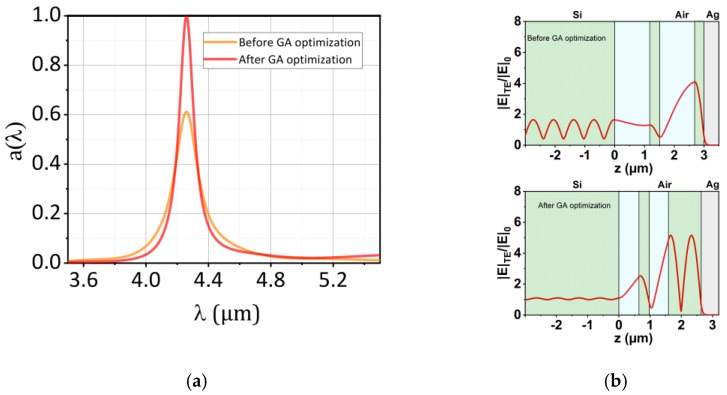
(**a**) Spectral response of the 1D TP structure (TE) before and after GA optimization at an inclination angle $\theta_{\beta }=25{}^{\circ}$ corresponding to a slab mode for a slab featuring w = 1 µm; (**b**) Normalized electric field before and after GA optimization

In order to start the constrained GA optimization process, a suitable start configuration is needed, as a pure DBR structure composed of four or six layers features low performance (low as explained in Section 3.1) as can be seen by the orange solid line in Figure 2a in case of the four layer configuration. Halving the air gaps (i.e., $d_{1}$ and $d_{3}$ in Figure 1a) results in a dielectric reflector (DR) with significantly reduced reflection at resonance. An additional measure for the four-layer configuration was tripling the width of the last QW Si layer $d_{\mathrm{last}}$. This procedure increases the fraction of the field enhancement inside the silicon and aligns the optical path lengths of the four and six layer configurations to a similar value. However, the reduction of the widths of the air gaps blue-shifts the resonance frequency. Therefore, the air layers feature a thickness of $\frac{\lambda_{\mathrm{eff}}}{8}$ and the Si-layers a thickness of $\frac{\lambda_{\mathrm{eff}}}{4n_{\mathrm{Si}}}$, respectively, where the effective wavelength $\lambda_{\mathrm{eff}}>\lambda_{0}$ has to be introduced in order to reach a resonance at $\lambda_{0}=4.26$ µm. This resulted in $\lambda_{\mathrm{eff}}=4.47$ µm in case of four ($\mathrm{Si}-d_{1}d_{2}d_{3}d_{\mathrm{last}}-\mathrm{Ag}$) and in $\lambda_{\mathrm{eff}}=5.45$ µm in case of six dielectric layers ($\mathrm{Si}-d_{1}d_{2}d_{3}d_{4}d_{5}d_{\mathrm{last}}-\mathrm{Ag}$), respectively.

**Figure A1:**
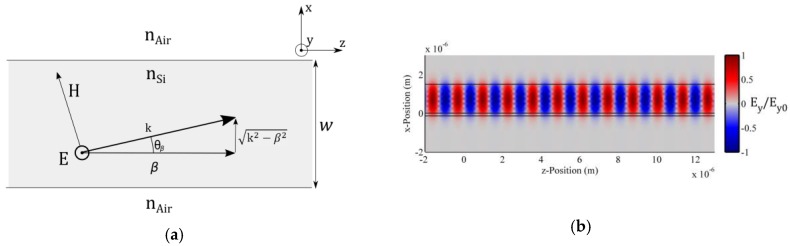
(**a**) Orientation of electric (E) and magnetic (H) fields for TE-polarization (**b**) Normalized electric field component $E_{y}\;$/$E_{y0}$ for a Si-slab waveguide with an additional ${\mathrm{Si}}_{3}N_{4}$ membrane with 140 nm thickness and w=1.5 µm

For the configurations with four and six dielectric layers this procedure results in sequences of layer thicknesses ($d_{1},\ldots ,d_{\mathrm{last}}$) ${\vec{d}}_{INI,\;4\;\mathrm{layers}}\;$ = (0.56, 0.32, 0.56, 0.93) µm and ${\vec{d}}_{INI,\;6\;\mathrm{layers}}\;$= (0.34, 0.39, 0.34, 0.39, 0.34, 0.37) µm, respectively, which provide a high emittance of ~0.9 at resonance and normal incidence. As the layered structure should be transformed into a slab scenario, the resonance should occur not only at the target $\lambda_{0}$, but also at the correct angle of incidence corresponding to the propagation constant of the guided mode associated with the Si-slab of height w (see Figure 2a). Thus, the vertical component $\vec{k}\cdot \sin\left(\theta \right)$ in the plane wave scenario should match the propagation constant β of a guided mode in the slab scenario, as explained in Appendix A.1. The propagation constant β together with the corresponding inclination angle $\theta_{\beta }$ can be calculated for a continuous slab waveguide (see Figure A1) from the characteristic equation for slab-modes. This yields values for β ($\lambda_{0}=4.26\;µm$) of 44735, 47107, 48486, and 49354 ${\mathrm{cm}}^{-1}$ for a Si slab with w= 0.75, 1, 1.25, and 1.50 µm, respectively. The translates to values for $\theta_{\beta }$ of 30.64°, 25.04°,21.17° and 18.34°. The initial configuration ${\vec{d}}_{INI,\;4\;\mathrm{layers}}$ and ${\vec{d}}_{INI,\;6\;\mathrm{layers}}\;$ can now be scaled with the factor $s_{w}\;=\frac{n_{\mathrm{Si}}}{n_{\mathrm{eff}}\left(w\right)}$, where $n_{\mathrm{eff}}\left(w\right)=\frac{\beta \left(w\right)}{k_{0}}$ is the effective index of the guided slab mode. 

These thicknesses constitute suitable starting configurations for the micro-GA optimization algorithm, which is able to enhance the emittance to unity at resonance together with a narrowing of the bandwidth For the GA optimization process, tight bounds for the thicknesses were employed (within 0.2 µm) in order to avoid parasitic resonances and facilitate slab confinement. The fitness function in dependence of the layer thicknesses $d_{i}$ was established in a similar way as in [17] and is minimized during the micro-GA algorithm

(5) $$F_{\theta_{\beta }}\left(d_{i}\right)=\sqrt{1-\epsilon_{1D}\left(d_{i},\theta_{\beta }\right)}\cdot \left(1+\int_{\theta_{\beta }-\delta \theta_{-}}^{\theta_{\beta }+\delta \theta_{+}}\epsilon_{1D}\left(d_{i},\;\delta \theta \right)\;d\left(\delta \theta \right)\right)$$

**Figure 3:**
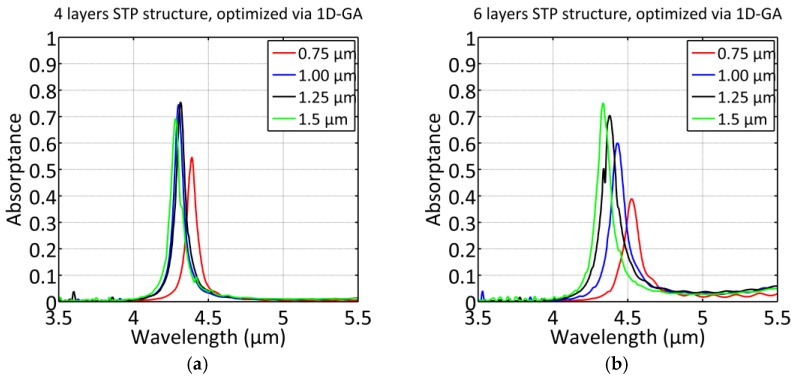
Spectral response of the STP structures (TE) for four (**a**) and six layers (**b**), respectively. The thicknesses were obtained by the GA optimization process featuring purely 1D TP structures.

In contrast to the previous fitness function employed in Ref. [16] this function optimizes both peak absorption (first factor) as well as the integrated angular distribution of the emittance around $\theta_{\beta }$ with $\left|\delta \theta_{+}+\delta \theta_{-}\right|=90{}^{\circ}$ (second factor). The latter directly correlates with narrowband emittance. This fitness function facilitates is able to find suitable configurations by applying tight upper and lower bounds to the initial configurations of the optimization process (i.e., $s_{w}\cdot {\vec{d}}_{INI,\;4\;\mathrm{layers}}\pm 150\;\mathrm{nm}$ and $s_{w}\cdot {\vec{d}}_{INI,\;6\;\mathrm{layers}}\pm 150\;\mathrm{nm}$, respectively). The optimization process was repeated 10 times and the configuration with the best score based on Equation (5) was chosen as initial configuration for the STP optimizations and shown in Table 1 and Table 2. We label this optimization procedure as “1D GA optimization” as the fitness function is calculated in a one-dimensional plane wave scenario. Figure 2a shows the achieved enhancement and narrowing of the absorption spectra and Figure 2b the enhancement of the normalized electric field for w=1 µm and N=4. Figure 3 shows the relative power absorbed by the silver in the STP geometry if the lateral dimensions obtained from the 1D optimization for every w are directly taken over to the slab scenario. It turned out that the obtained configurations served as a good starting point for the GA optimization featuring a fitness function in the STP scenario. The results are discussed in more detail in Section 5.

## 4. Optimization Using STP Structures as Fitness Function

Although the method of optimizing the lateral dimensions of the STP structures via simulations in the 1D domain successfully yields resonant absorption and fast calculation, it cannot deliver accurate optimum results due to fundamental differences between 1D TP and STP structures. For example, the STP modes feature different absorptive properties because of the introduction of radiation damping and reduced metal damping due to the modified STP mode shape. As discussed in Section 2, the dominating power loss for a high *Q* STP resonance (i.e., $Q_{P}=Q_{\mathrm{rad}}\gg Q$) is not the reflection of the wave back to the port, but the spurious radiation. As a result, high *Q* resonances in the 1D domain are not able to yield an optimal resonance condition for the STP structures. Thus, further optimization of the lateral dimensions is necessary for the slab scenario. There are many approaches to optimize the resonance of photonic crystal slab structures in order to maximize Q factor and intensity of the resonance, see, e.g. [18,26,27]. However, the methods were only applied to purely dielectric structures rather than hybrid metal-dielectric TP structures. Despite the presence of metal causing an inherent asymmetry in the field distribution across the interface between Bragg mirror and metal, some of these concepts, such as optimization of momentum space design or *Q*-factor cancellation, can potentially be also applied to TP structures. 

Here we apply a second time a GA optimization algorithm using a fitness function in the slab scenario, which calculates the time-averaged net power flux into the bulk silver $P_{Z}^{\mathrm{Ag}}$

(6) Fβ(di)=1−PZAgPin =1+12∫0wRe(Ey(zint,x)⋅Hx*(zint,x)) dx

The incident power from the port $P_{\mathrm{in}}$ is set to 1 W/m and the term $\frac{P_{Z}^{\mathrm{Ag}}}{P_{\mathrm{in}}}$ in (6) corresponds to the absorptance $A_{\mathrm{STP}}$. The validity of Kirchhoff’s law for the STP structures is discussed in Appendix A.3. In contrast to the 1D fitness function (5) featuring integration over many angles, the calculation of $A_{\mathrm{STP}}\left(\lambda_{0}\right)$ had to be performed only once per individual and generation for an incoming guided mode with $\beta_{0}$ corresponding to a vacuum wavelength $\lambda_{0}=4.26$ µm. Hence, $F_{\beta }$. only maximizes the field enhancement inside the STP structure, i.e., coupling of the guided mode to the STP resonance and does not directly maximize the resonance *Q*-factor. However, as this approach does minimize the radiation loss, the resonance *Q*-factor is also optimized indirectly, as can be seen from (4). The GA optimization featured variations of the layer thicknesses $d_{i}$ within tight bounds of ≈200 nm. The upper boundary was taken from the corresponding thickness value of the preceding one-dimensional GA optimization. This value can be taken as upper bound, as the redshift of the resonant wavelength due to the electric field squeezing effect described in Appendix A.4. demands a decrease of the lateral dimensions if the resonant frequency should be kept constant. We label this procedure as “STP GA optimization”, as the fitness function (6) was calculated in the two-dimensional slab scenario. The population size in the µ-GA process was set to 8 for the four-layer configurations and to 10 for the six-layer configurations. 

**Figure 4:**
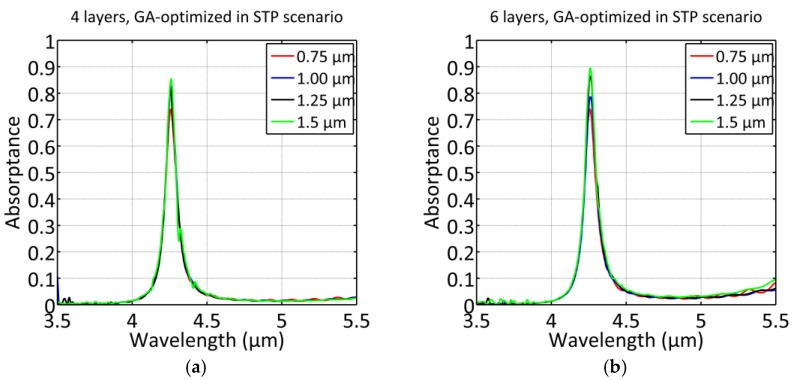
Spectral response of the STP structures (TE) for four (**a**) and six layers (**b**), respectively. The thicknesses were obtained by the GA optimization process featuring slab-TP structures.

Figure 4 shows the relative power absorbed by the silver in the STP geometry if the lateral dimensions are obtained with (6) as fitness function. The configurations obtained via the fitness function (5) constituted the start configurations for initializing the optimization process.

## 5. Results and Discussion

### 5.1. STP Structures via 1D GA Optimization 

#### 5.1.1. Comparison between STP and 1D TP Resonances

Table 1 and Table 2 state the peak absorptance associated with the Ag surface $A_{\mathrm{STP}}\left(\lambda_{0}\right)$ of the STP structures with N = 4 and N= 6, respectively, and optimized via fitness function (5) using purely one-dimensional TP structures. Thus, the lateral dimensions of the layers obtained by the 1D TP optimization are directly applied to the slab scenario without subsequent scaling. It is interesting to observe a distinct improvement of the resonance *Q*-factor ($=\frac{\lambda_{0}}{\mathrm{fwhm}}$, where fwhm is the full with at half maximum of the absorption spectrum) for the slab structures ($Q_{\mathrm{STP}}$) compared to the quality factor of the 1D TP structures ($Q_{1D}$). Initially, it could be assumed that the radiation loss lead to a decrease of the resonance *Q-*factor. 

**Figure 5:**
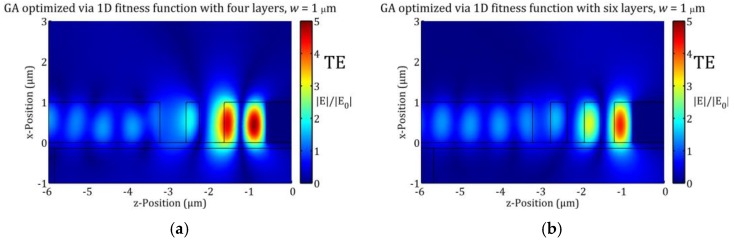
Resonance profile showing the relative field enhancement |E|/$\left|E_{0}\right|$ ($E_{0}$ denotes electric field without TP structure) for (**a**) four layers (two etched air gaps) and (**b**) six layers (three etched air gaps) for TE polarization and w = 1 µm. The fields correspond to thickness configuration obtained via GA optimization featuring 1D TP structures (see Table 1). The higher electric field in panel (**a**) leads to a higher absorptance by silver of 75%, whereas in panel (**b**) the field corresponds to an absorptance of only 60%.

However, this assumption is misleading as the radiation loss rate is small and the resonance damping is already dominated by leakage into the WG and the metal. Consequently, the observed *Q*-factor improvement should be linked to the field distribution near the Ag surface resulting from STP-mode at resonance. The intensity of the electric field next to the Si-Ag interface is varying substantially in dependence of the x-position (i.e., slab height), in contrast to the 1D TP structures featuring uniform electric field amplitudes on planes inside the metal and parallel to the interface. Thus, the shape of the E-field profile (see Figure 5a,b) determines the resonance damping associated with the metal. As a result, the damping rates of the slab resonance are significantly reduced leading to a higher *Q*-factor of the resonance. More specifically, switching from the 1D TP structure to a STP structure particularly leads to a significantly higher value of $Q_{\mathrm{LM}}$ at resonance. Increasing radiation loss (i.e., reducing $Q_{\mathrm{rad}}$) seems to compensate in part the reduced field-metal interaction area for thinner slabs partly preserving the resonance condition reflected by Equation (4), as decent coupling into the STP mode is observed (55%/40% absorption for four/six layers, respectively, and w=0.75 µm) for the lateral dimensions obtained with the 1D optimization.

#### 5.1.2. Comparison between Configurations with Four and Six Layers

Another interesting feature revealed in this study are significantly lower values for $Q_{\mathrm{STP}}$ as well as for $Q_{1D}$ for the six layer configurations (*N* = 6) compared to the four layer configurations (*N* = 4). Particularly, the decrease of $Q_{1D}$ may seem to contradict the results in [16], where an increase of *N* allowed the GA optimization process a dramatic narrowing of the spectral bandwidth. The reason for this seeming contradiction is that the configurations in Table 1 and Table 2 were derived via a constrained GA optimization process featuring bounds on the layer thicknesses. It was already shown in [17] that increasing the number of layers does not necessarily lead to an improvement of the resonance quality factor. This characteristic difference between *N* = 6 and *N* = 4 does continue (in a less pronounced manner) for the structures optimized via STP structures (see below in Section 5.2.). On the other hand, the values of $A_{\mathrm{STP}}\left(\lambda_{0}\right)$ are fairly similar, but the structures with *N* = 6 revealed monotonically increasing behavior with the slab thickness w, in contrast to the structures with *N* = 4. Thus, the wider Si layer next to the Ag surface ($d_{\mathrm{last}}>1$ µm) for the configurations with *N =* 4 facilitates a greater light confinement (i.e., higher *Q*-factor). Despite the structures with *N* = 6 feature a similar coupling to the TP modes (i.e., similar values for $A_{\mathrm{STP}}\left(\lambda_{0}\right)$), the enhanced coupling to radiation results into a worsened value of $Q_{\mathrm{STP}}$. This is indicated by Figure 5a,b by comparing the relative electric field enhancements between both panels.

#### 5.1.3. Absorptance by Silver in Dependence of Slab Thickness

It can be seen that the absorption performance is generally increasing for increasing slab height, with the exception of the four-layer configuration with w= 1.5 µm, where the peak performance drops in comparison to the configuration with 1.25 µm. The latter features an excellent value of $A_{\mathrm{STP}}\left(\lambda_{0}\right)$ of 75% reflecting a very good fulfilment of the resonance condition for these configurations. The impaired resonance condition for the former structure with w= 1.5 µm results in increased reflection back into the waveguide. Also, it can be seen from Table 1 and Table 2, that $Q_{\mathrm{STP}}$ does not depend on the slab height w in an obviously systematic manner.

This can also be interpreted by the consideration described in Section 5.1.1. that radiation modes are able to compensate parts of the decreasing leakage losses into the waveguide (characterized by increasing $Q_{\mathrm{LM}}$) for decreasing w. The slab height is the crucial parameter for an efficient compensation and coupling into the STP mode, although the optimum cannot be achieved with the GA optimization process using 1D TP structures. For the configurations with *N* = 4 (see Table 1), the slab height for optimal compensation is exceeded at w=1.5 µm, resulting into increased reflection back into the waveguide and into a drop of $A_{\mathrm{STP}}\left(\lambda_{0}\right)$ as well of $Q_{\mathrm{STP}}$. Although the progression of the absorption functions $A_{\mathrm{STP}}\left(\lambda_{0}\right)$ in Figure 3b seemingly indicates a saturating behavior, a further increase of w would gradually lead to an effective plane wave scenario (Figure 2a,b). The correlation between the adjacent functions (differing in w) relates to the rather small modifications in lateral thicknesses from $d_{1}$ to $d_{5}$, which can be seen in Table 1.

### 5.2. GA Optimized Configurations Featuring STP Structures

**Figure 6:**
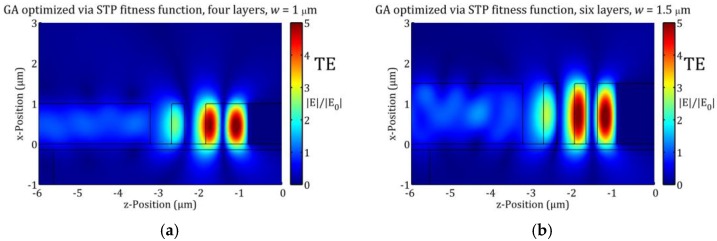
Resonance profile showing the relative field enhancement |E|/$\left|E_{0}\right|$ ($E_{0}$ denotes electric field without TP structure) for (**a**) four layers (two etched air gaps) with w = 1 µm and (**b**) six layers (three etched air gaps) with w = 1.5 µm. The fields correspond to thickness configuration obtained via GA optimization featuring 1D TP structures (see Table 2). The higher electric field in panel (a) leads to an absorptance by silver of 82%, whereas in panel (b) the field corresponds to a peak absorptance of 90%.

Figure 6 and the Table 3 and Table 4 reveal significant improvements by applying the GA optimization using STP structures in terms of absorptance for all number of layers *N* and slab thicknesses w. This second optimization step allows an adequate fine-tuning to achieve the best fulfillment of the resonance condition for TP structures possible. However, it can be seen that some trends from the 1D GA optimized structures can be transferred to the STP-GA optimized configurations. The structures with *N* = 4 feature excellent absorption for the thinner slabs (0.75, 1 µm), whereas the best absorptances are achieved by the structures with *N* = 6 feature thick slabs (1.25, 1.5 µm). Interestingly, also the Q- factors remain at a slightly higher level for the four-layer configurations compared to the six-layer configurations. This reflects the high confinement of the electric field inside the wider layer $d_{\mathrm{last}}\approx 3\cdot \frac{\lambda }{4n_{Si}}$ µm, but the few layers preceding this layer exacerbate optimal coupling to the TP resonance for the thicker slabs. As a result, the six-layer configurations can provide a better leakage (lower $Q_{\mathrm{LM}}$) of the STP resonance for the thicker slabs, which reduces reflection and suppresses radiative losses to an optimal level. It is also interesting to look at the progression of the air gap $d_{5}$ in Table 4 for decreasing w, the GA-algorithm optimization results in decreasing $d_{5}$ down to 0.18 µm, which represents a convergence to the composition of the corresponding four-layer configurations. The lower bound on $d_{5}$ of the constraint GA-optimization prevents a further decrease for w=0.75 µm. 

Up to 90% relative absorption is achieved for six dielectric layers and w = 1.5 µm, which demonstrates the potential for resonant slab structures for various applications. However, thick slabs come at the cost of a higher mode volume of the waveguide, which causes losses to mode coupling. Also, the evanescent field gets negligible, which represents a downside in case evanescent field absorption sensors. A scenario that benefits from thicker slabs (e.g. $w\approx \frac{\lambda_{0}}{n}$) would be slab- or pillar-photonic crystal (PC) waveguides, for example. A pure slab mode can be coupled via optimized tapers to a rib/slab-PC waveguide rather efficiently [28,29].

### 5.3. Conclusion and Outlook

The STP structures found by the two-step optimization process revealed resonances featuring surprisingly well confined electric fields and high metal absorptance even for thin slab structures. This can enable novel monolithically integrated, optical sensor devices with higher sensitivity compared to similar integrated devices featuring non-resonant structures. A reliable prediction of the power levels that can be expected heavily depends on the temperatures that can be reached until thermal stress between metal and dielectrics will lead to failure of the device. However, assuming that temperatures of 700–800 K are possible, a spectral radiation density of 2–5 nW/(µm^2^ µm rad) in the slab can be expected. This number should scale linearly with the width of the slab. This estimation follows from the radiation spectrum of an ideal blackbody (Planck’s law of radiation) multiplied with the spectral functions for the emittance calculated in this work.

Although we focused on the application as a thermal emitter by using the appropriate material parameters at elevated temperatures in this work, STP structures also have huge potential for integrated absorber structures especially in combination with pyroelectric materials, for example. Also, the application as an absorber has the advantage of more exact simulations compared to emitters, as material parameters in literature are more reliable at room temperatures. Especially, the combination of STP emitter and absorber structures have potential to enhance the detection limit significantly for integrated devices.

A crucial limitation for the emission intensity will be constituted by the maximum operation temperature for an STP-emitter. The hybrid metal-dielectric junctions naturally will cause mechanical stress on the membrane due to the different coefficients of thermal expansion. Also, disintegration of metal may deteriorate the metal–dielectric interface and lead to failure of the device.

## Figures and Tables

**Table 1:** Widths, relative absorption, and quality factors for the obtained GA optimized configurations using 1D TP structures and four dielectric layers with a target resonance wavelength $\lambda_{0}=4.26$ µm. They differ in the target inclination angle $\theta_{\beta }$ corresponding to different slab thicknesses in the waveguide-scenario.

w (µm)	$d_{1}$	$d_{2}$	$d_{3}$	$d_{\mathrm{last}}$	$A_{\mathrm{STP}}\left(\lambda_{0}\right)$	$Q_{1D}$	$Q_{\mathrm{STP}}$
1.5	0.49	0.35	0.58	1.00	69 %	36	49
1.25	0.64	0.30	0.62	1.03	75 %	36	60
1	0.64	0.33	0.62	1.05	75 %	38	59
0.75	0.69	0.43	0.63	1.12	55 %	37	55

**Table 2:** Configurations featuring six dielectric layers, analogous to Table 1

w (µm)	$d_{1}$	$d_{2}$	$d_{3}$	$d_{4}$	$d_{5}$	$d_{\mathrm{last}}$	$A_{\mathrm{STP}}\left(\lambda_{0}\right)$	$Q_{1D}$	$Q_{\mathrm{STP}}$
1.5	0.43	0.4	0.36	0.39	0.26	0.445	75 %	28	39
1.25	0.31	0.42	0.47	0.34	0.38	0.43	70 %	24.5	36
1	0.44	0.42	0.42	0.39	0.35	0.46	60 %	27	38
0.75	0.54	0.43	0.48	0.46	0.34	0.49	39 %	28	36

**Table 3:** Widths, relative absorption, and quality factors for the obtained GA optimized configurations using the fitness function (6) featuring STP structures. Four layers were varied for finding high fitness configurations at the target resonance wavelength $\lambda_{0}=4.26$ µm.

w (µm)	$d_{1}$	$d_{2}$	$d_{3}$	$d_{\mathrm{last}}$	$A_{\mathrm{STP}}\left(\lambda_{0}\right)$	$Q_{\mathrm{STP}}$
1.5	0.58	0.31	0.41	1.02	85%	54
1.25	0.62	0.26	0.59	1.02	83%	50
1	0.53	0.32	0.52	1.06	82%	51
0.75	0.47	0.37	0.53	1.10	74%	46

**Table 4:** Six dielectric layers, analogous to Table 3

w (µm)	$d_{1}$	$d_{2}$	$d_{3}$	$d_{4}$	$d_{5}$	$d_{\mathrm{last}}$	$A_{\mathrm{STP}}\left(\lambda_{0}\right)$	$Q_{\mathrm{STP}}$
1.5	0.51	0.33	0.43	0.28	0.28	0.49	90%	51
1.25	0.46	0.35	0.47	0.30	0.23	0.51	86%	45
1	0.45	0.33	0.54	0.34	0.19	0.53	79%	45
0.75	0.45	0.36	0.53	0.38	0.18	0.55	73%	43

## Appendix A

### Appendix A.1. Properties of Si Slab Waveguide Modes and Polarization

As the incident and reflected waves of the STP structures are represented by guided slab modes, we need to study how their properties impact the STP resonance. Although slab waveguides support TE as well as TM modes, they differ significantly in their dispersion relation, as TM modes featuring the same propagation constant β carry more energy [19]. At first thought, this would simply result in different resonant wavelength for TE and TM as consequence for the STP resonators.

However, the TM mode does not couple to a STP resonance for the following reasons: First, the in-plane electric field component $E_{z}$ is features an odd profile with respect to the plane $x=\frac{w}{2}$ [19]. This results into enhanced coupling to radiation. Second, the STP structure, particularly the metal interface, is highly anisotropic due to the infinite out-of-plane dimension (*y*-direction) and the severely limited vertical dimension (*x*-direction) of the slab. As a consequence, the absorption and emission behavior is expected to be highly polarization dependent, as demonstrated in [12] for TP structures. As a matter of fact, we did not observe coupling into the STP resonator structures for incident TM slab modes. The even $E_{y}$ profile for TE polarization is shown in Figure A1b.

Figure A1a depicts the situation for a simple Si slab waveguide guided TE mode with air in the cladding and substrate region. The resonance of the STP structure is determined for the most part by the propagation constant β, which corresponds to an inclination angle $\theta_{\beta }=\arctan\left(\frac{\beta }{\sqrt{k^{2}-\beta^{2}}}\right)$ (see Figure A1, k denotes the total wave vector in Si) and can be calculated in a straight forward way by the characteristic equation for slab waveguides [20]. Although the STP and 1D TP structures differ substantially in their properties, the spectral response of the STP structure can be approximated by the 1D TP simulations by applying the corresponding $\theta_{\beta }$ as incident angle of the incoming plane wave. Thus, $\theta_{\beta }$ for every slab height w is calculated for the simulations in the 1D domain in order to be used as incident angle of the incoming plane wave.

### Appendix A.2. Simulation Methods of 1D and STP Domain

For the 1D TP structure (see Figure 1a) the Transfer-Matrix method was employed, which offers excellent computation speed for GA optimization processes [16,17,30]. In contrast, the STP scenario requires a more complex 2D simulation domain, as can be seen in Figure 1b.

In the latter case FEM is a suitable method for steady-state problems, as it offers high computation speed by varying the precision over the simulation domain. Particularly, a finer mesh was required at the Si-Ag interface for correct evaluation of the absorbed power fraction of the Ag particle via Poynting’s theorem. The mesh size had to be reduced during the GA optimization processes in order to achieve a feasible computation time, as meshes have to be recalculated for every individual member of the population of stack configurations. Although this decreases the evaluated power absorbed of the silver particle because of the more inaccurate resolution of the Si-Ag interface, the global maxima obtained from the optimization process did not change compared to a finer mesh resulting from a finalized mesh convergence analysis. The finer mesh was used for calculating the full spectra shown in Figure 4a,b and Figure 5a,b, respectively.

Utilizing Kirchhoff’s law, an incident TE polarized wave is launched in the 1D TP as well in the STP scenario. In the former 1D case, this was achieved by a simple plane wave propagating in positive *z*-direction (i.e., oscillating amplitude proportional to $e^{+i\left(kz-\omega t\right)}$), whereas in the STP scenario the fundamental guided slab mode was calculated at the port boundary (see Figure 1b) via a *Boundary Mode Analysis* of the software package Comsol Multiphysics 5.3 (Version Number, COMSOL Inc., Stockholm, Sweden). Absorbing boundaries (i.e., second order “scattering boundary conditions”) were applied at the side walls of the simulation domain.

With Kirchhoff’s law and the fact that there is no transmission for TP-emitters featuring DBR-side emission, the emittance ϵ and the absorptance *A* for the 1D-TP structures can be evaluated as $\epsilon_{1D}=A_{1D}=1-R$, where R is the reflectance of the structure. This approach allows efficient simulation of one-dimensional plasmonic emitter structures. As there are scattering losses for the slab structures, this cannot be directly applied to the STP scenario. In this case, R can only determine the total power absorbed in the simulation domain $A_{\mathrm{total}}$, not the power absorbed only by the Si-Ag interface $A_{\mathrm{STP}}$. As the latter can be calculated by Poynting’s theorem (as mentioned above), this raises the question whether Kirchhoff’s law still holds for the STP structures (i.e., $\epsilon_{\mathrm{STP}}=A_{\mathrm{STP}}$). This question is discussed in Appendix A.3.

### Appendix A.3. Validity of Kirchhoff’s Law and Reciprocity

As described in Appendix A.2, we utilize Kirchhoff’s law and reciprocity by simulating the reciprocal situation of an incident TE-polarized wave (propagating in positive z-direction) for the 1D as well as for the STP scenario. Considering STP structures, the power absorbed by the silver is calculated via Poynting’s theorem, in particular by integration of the time-averaged net power flux (Poynting vector) along the Si-Ag interface. Intuitively, the time-reversibility of the STP-absorption process is violated due to radiative and intrinsic material losses. Fortunately, theoretical works showed that time-reversibility is not a necessary condition for the validity of Kirchhoff’s law. The only condition necessary for the validity is reciprocity of the bidirectional reflection distribution function (BRDF). A general definition of the BRDF in the framework of statistical optics was derived in [31] together with a general proof of Kirchhoff’s law. Thus, the validity of Kirchhoff’s law was proven for many types of interfaces including highly-scattering semi-infinite media. In [32], reciprocity was proven for vector wave fields containing evanescent components via Lorentz’s reciprocity with dipole sources at a finite distance from the scatterer. As a consequence of Lorentz’s reciprocity and Ref. [32], reciprocity relations can be derived even for time irreversible processes (i.e., absorption processes featuring scatterers with losses). With these considerations, it can be expected that the coupling efficiencies between a slab waveguide mode and a STP mode satisfy reciprocity, which leads to validity of Kirchhoff’s law for STP structures by [31]. As a result, the absorptance evaluated by Poynting’s theorem $A_{\mathrm{STP}}$ is expected to correspond to the emittance of the STP-structure $\epsilon_{\mathrm{STP}}$, just as for the one-dimensional scenario.

### Appendix A.4. The Effect of Limiting the Extent of the Elctric Field Vertically

The resonance frequency of the STP structure does not exactly correspond to the resonance frequency of the 1D TP structure at an incident angle $\theta_{\beta }$. The reason for this is the hybrid localization in two directions of the resonant mode. Similarly to a rectangular waveguide scenario, the shape of the field in the corner regions changes the energy stored in the resonant mode. Figure 5 and Figure 6 depict the electric field profiles of the STP structures at resonance, which demonstrate a significant deviation from an ideal 1D field distribution. The more the electric field profile is vertically “squeezed” inside the slab (i.e., approaches the cut-off frequency of the waveguide), the more pronounced is the change in energy. This modification of the resonance energy can be quantified with a perturbation approach or the effective index method, as utilized for the theoretical description of guided modes of waveguides with rectangular cross-section in, e.g., [20].

The shift of resonance frequency demands a rescaling of layer widths of the STP structures for achieving a particularly targeted resonance wavelength $\lambda_{0}$. Reducing w increases the ‘squeezing’ of the electric field and counteracts the increase of the lateral dimensions due to the subsequent decrease of β. Furthermore, the vertical squeezing of the electric field mode profile also impacts the coupling to radiation modes as well as the peak absorptance $A_{\mathrm{STP}}\left(\lambda_{0}\right)$. The former can be exemplified by more pronounced Fourier components inside the light cone for reduced β (or reduced slab thickness w), as the vertical component of the wave vector $k\sin\left(\theta_{\beta }\right)$ is increasing. The change of the absorption properties associated to the TP resonance results from the increasing non-uniformity of the electric field amplitude with respect to the vertical (x-) direction associated with the mode shape, which, when compared to the 1D case, leads to a non-uniform field distribution at the silver surface. This reduction of the absorption by silver impacts the *Q* of the STP resonances, as discussed in Section 5.
